# Persistent cough after segmental resection, an issue that clinicians need to pay more attention to

**DOI:** 10.3389/fonc.2025.1621841

**Published:** 2025-08-08

**Authors:** He Guan, Zhijun Han, Qifei Wan, Qiangwen Huang, Li Wei, Jiwei Li

**Affiliations:** ^1^ Department of Thoracic Surgery, Zhengzhou University People's Hospital, Henan Provincial People's Clinical Medical School of Zhengzhou University, Zhengzhou, Henan, China; ^2^ Department of Thoracic Surgery, Henan Provincial People’s Hospital, Zhengzhou, Henan, China; ^3^ Department of Thoracic Surgery, Henan University People’s Hospital, Zhengzhou, Henan, China

**Keywords:** thoracoscopic surgery, segmentectomy, cough, prediction model, nomogram

## Abstract

**Introduction:**

Persistent cough after pulmonary resection can significantly impair a patient's quality of life. However, risk factors for persistent cough after pulmonary segmentectomy remain insufficiently described. This study aims to explore the risk factors for persistent cough following pulmonary segmentectomy and to establish a predictive model to guide perioperative management.

**Methods:**

This retrospective study included 540 patients who underwent thoracoscopic pulmonary segmentectomy at Zhengzhou University People’s Hospital from February 2024 to January 2025. Data were divided into training and internal validation cohorts. Multivariate logistic regression analysis was performed using the training cohort to develop a predictive model. Data from 160 patients who underwent thoracoscopic pulmonary segmentectomy at Zhengzhou Seventh People’s Hospital from August 2024 to February 2025 were used for external validation. Both validation cohorts were used to evaluate the model’s reliability and its impact on patient outcomes.

**Results:**

There were no significant differences in the distribution of variables between the training and internal validation cohorts. Tobacco exposure (OR 0.19, 95% CI 0.07-0.49), tumor location (OR 2.36, 95% CI 1.27-4.36), type of surgery (OR 3.70, 95% CI 2.18-6.29), vagus nerve injury (OR 2.15, 95% CI 1.23-3.76), and drainage duration (OR 1.48, 95% CI 1.29-1.70) were identified as independent risk factors for persistent cough after surgery. The predictive model derived from multivariate analysis demonstrated high diagnostic value (AUC = 0.80), and the Hosmer-Lemeshow test indicated good model fit (P = 0.818). Both internal and external validation analyses confirmed the model’s reliability and substantial net benefit for patients.

**Discussion:**

Persistent cough is a common postoperative complication following pulmonary segmentectomy and should receive greater clinical attention. Tobacco exposure, tumor location, type of surgery, vagus nerve injury, and drainage duration were identified as independent risk factors for persistent cough after surgery. Visualizing these risk factors aids in assessing the likelihood of persistent cough after pulmonary segmentectomy and provides valuable support for clinical decision-making.

## 1 Introduction

Non-small cell lung cancer (NSCLC) remains one of the most prevalent and lethal malignancies globally, posing significant health and economic burdens (1). With the development of imaging technologies and increased health awareness, early diagnosis rates for lung cancer have improved in recent years (2). Studies have shown that for NSCLC patients with tumor diameters ≤2 cm and consolidation tumor ratio between 0.5–1, there are no significant differences in complications, mortality, or 5-year overall survival between segmentectomy and lobectomy (3). Additionally, segmentectomy preserves more lung tissue and is generally better accepted by patients (4), making it the preferred treatment for many early-stage NSCLC cases.

Persistent cough after pulmonary resection is a common complication following lung cancer surgery, with an incidence ranging from 18% to 50% (5, 6). This condition can lead to fatigue, insomnia, spontaneous pneumothorax, and other complications (7). Furthermore, postoperative persistent cough may increase psychological burden, causing patients to question the efficacy of the surgery and leading to anxiety and depression, ultimately reducing quality of life (8–10). However, compared to life-threatening complications such as chylothorax, pneumothorax, or progressive hemothorax, persistent cough is often considered a secondary symptom and is only reported when it severely impacts daily life and work, leading to it being frequently overlooked by clinicians.

Preoperative pulmonary rehabilitation and preventive treatment have been shown to reduce the incidence of persistent cough after pulmonary surgery (11–13). Identifying high-risk patients early and implementing interventions to reduce the risk of postoperative cough and subsequent healthcare costs is a critical clinical challenge and an essential component of promoting faster patient recovery.

Currently, there is limited research specifically addressing the risk factors for persistent cough following pulmonary segmentectomy, and existing studies on this topic are somewhat controversial. Therefore, this study aims to investigate the risk factors for persistent cough after pulmonary segmentectomy and to establish a visual predictive model that will provide new evidence for clinicians to prevent and manage this complication.

## 2 Materials and methods

### 2.1 Study design and population

This study was a retrospective study approved by the Ethics Committee of Zhengzhou University and the Ethics Committee of the Seventh People’s Hospital of Zhengzhou. All methods were conducted in accordance with relevant regulations.

Patients undergoing thoracoscopic segmentectomy for benign or malignant pulmonary lesions were included as study subjects. The diagnostic criteria for postoperative persistent cough were as follows: exclusion of postnasal drip syndrome, bronchial asthma, and oral administration of angiotensin-converting enzyme inhibitors, along with a dry cough persisting for ≥2 weeks post-surgery without significant abnormalities on chest X-ray (5).The exclusion criteria were as follows: (a) Pre-existing respiratory diseases or a history of chronic cough; (b) A history of thoracic or pulmonary surgery or malignancy; (c) Preoperative or postoperative use of medications that may induce cough; (d) Requirement for reoperation due to complications such as progressive hemothorax; (e) Conversion to open thoracotomy during surgery; (f) Loss to follow-up or incomplete clinical data; (g) Patients who received neoadjuvant therapy or adjuvant therapy; (h) Failure to meet the predefined study protocol. (The patients selection details are provided in the **Supplementary Materials**).

### 2.2 Data collection

Patient data were collected from February 2024 to January 2025 at Zhengzhou University People’s Hospital and from August 2024 to February 2025 at the Seventh People’s Hospital of Zhengzhou. The baseline data included name, sex, age, BMI, history of underlying diseases (The underlying diseases are strictly limited to chronic diseases that are not contraindicated for surgery. The specific conditions include: hypertension, coronary heart disease, diabetes, old cerebral infarction, peptic ulcer, etc.), and tobacco exposure.

All patients underwent preoperative examinations at our institution, from which we collected tumor location and pulmonary function data. In addition, surgical records and hospitalization details were reviewed to obtain information on surgical site, type of surgy, lymph node dissection, and vagus nerve injury. Based on tumor location, surgical procedures were classified into segmentectomy and combined segmentectomy. Combined segmentectomy was performed for lesions located at the boundaries of two or more pulmonary segments to ensure adequate surgical margins.

All patients underwent double-lumen endotracheal intubation under general anesthesia. Intraoperatively, resected specimens were subjected to frozen-section pathological examination. If confirmed as benign, the procedure was concluded without lymph node dissection; otherwise, mediastinal lymph node sampling (three N2 groups and one N1 group) was performed. Regional lymph node classification was based on the International Association for the Study of Lung Cancer (IASLC) lymph node map (14).

Postoperatively, all patients were monitored in the thoracic surgery department. A chest X-ray was performed on postoperative day 2. The chest tube was removed if the drainage volume was <200 mL/day and there was no evidence of air leakage, pneumothorax, or pleural effusion.

Patients attended routine outpatient follow-up visits 4 weeks after surgery, during which persistent postoperative cough was recorded in the electronic medical record. The severity of persistent postoperative cough was assessed using a self-designed numerical rating scale (NRS), where 0 indicates no cough, 1–3 indicates mild cough with no impact on sleep, 4–6 indicates moderate cough affecting sleep but not preventing it, 7–9 indicates severe cough preventing sleep or causing nighttime awakening, and 10 indicates intense, uncontrollable coughing.

The sample size calculation employed an automated method for determining the required sample size based on the R package “pmsampsize,” as detailed at https://mvansmeden.shinyapps.io/BeyondEPV/ (15). This package computes the sample size across four dimensions, adopting the maximum value as the requisite sample size: 1) calculating the sample size needed to accurately estimate the intercept in a model without predictor variables (null model); 2) estimating the sample size necessary to ensure that the new predictive model maintains a small average prediction error within the target population for binary outcomes; 3) determining the sample size and the number of candidate predictor parameters to ensure a shrinkage effect of the predictor variables (e.g. ≤10%), employing shrinkage methods to mitigate overfitting by reducing model prediction variability; 4) calculating the sample size to prevent overly optimistic model fitting to training data, thereby providing a more realistic estimate of model fit. The parameters used in the calculation include the number of predictors, the proportion of the expected outcome in the target population, and the root mean square error, which generally does not exceed 0.05. Building upon preliminary literature reviews (5, 6, 16), we conservatively overestimated the relevant parameters, assuming an overall complication incidence rate of 30% post-operation, with four main influencing factors and a root mean square error of 0.05. Utilizing these parameters, we estimated the required sample size for constructing a predictive model to be 370 cases.

### 2.3 Statistical processing and analysis

The dataset was randomly divided into a training cohort and a validation cohort. Continuous variables were presented as mean ± standard deviation. Independent sample t-tests and chi-square tests were used to compare clinical characteristics between the two cohorts. Statistical analyses were conducted using SPSS version 26.0 (IBM, Armonk, NY, USA), with statistical significance defined as P < 0.05.

### 2.4 Development and assessment of the nomogram

In the training cohort, univariate and multivariate logistic regression analyses were performed to identify factors associated with persistent postoperative cough. Multicollinearity was assessed using the “collinear” package in R software (version 4.3.2, Vienna, Austria). LASSO regression via the R software “glmnet” package.The final independent risk factors were incorporated into a nomogram constructed using R to predict the occurrence of postoperative cough following segmentectomy. Internal and external validation were performed using the validation cohorts.

Model calibration was assessed using the Hosmer-Lemeshow test. Predictive accuracy and agreement were evaluated using the receiver operating characteristic (ROC) curve, area under the curve (AUC), and calibration curve. Decision curve analysis (DCA) was conducted to assess the net clinical benefit of the model. The nomogram and calibration curves were generated using the “rms” package, ROC curve analysis was performed using the “pROC” package, and DCA was conducted using the “rmda” package in R.

## 3 Result

### 3.1 General characteristics and differences

A total of 540 patients were included in the training and internal validation cohorts. The mean age of the included patients was 55.96 years, with 344 (63.70%) being female. Among these patients, 346 (91.67%) had malignant tumors, while 34 (8.33%) had benign tumors. The overall incidence of persistent postoperative cough was 31.11% (n=168). Notably, 76.79% (n=129) of these cases presented as irritant dry cough, primarily triggered by strong odors or cold air. Most patients exhibited mild to moderate cough severity (the proportion of people with mild cough was 63.69%, n = 107; the proportion of people with moderate cough was 25%, n = 42).

The study population was randomly assigned into a training cohort (n=378) and internal validation cohort (n=162) at a 7:3 ratio. The details are presented in **Table 1**. There were no statistically significant differences in baseline characteristics between the training and internal validation cohorts (all P > 0.05), indicating that the randomization process was balanced and minimized potential selection bias. The comparison between the training cohort (n=378) and the external validation cohort (n=160) is shown in **Table 2**. Except for the difference in postoperative drainage time, there was no statistical difference in the data of other variables between the two cohorts.

**Table 1:** Baseline characteristics of all patients in the training cohort and internal validation cohort.

Variables		Training cohort (n=378) M (P25, P75)/N (%)	Internal validation cohort (n=162) M (P25, P75)/N (%)	t/χ^2^	P
Age		55.36 (49, 63)	57.35 (50, 65)	-1.80	0.073
BMI		23.90 (22.03, 25.81)	23.99 (22.00, 25.83)	-0.30	0.761
Drainage duration		3.63 (2, 5)	3.87 (2, 5)	-1.22	0.222
Sex	Male	137 (36.24)	59 (36.42)	0.01	0.969
	Female	241 (63.76)	103 (63.58)		
Underlying disease	–	253 (66.93)	100 (61.73)	1.36	0.244
	+	125 (33.07)	62 (38.27)		
Tobacco exposure	Never	297 (78.57)	116 (71.60)	3.06	0.195
	Former	9 (2.38)	4 (2.47)		
	Current	72 (19.05)	42 (25.93)		
Tumor location	UL	100 (24.46)	45 (27.78)	0.1	0.751
	NUL	278 (73.54)	117 (72.22)		
Type of surgery	Single	263 (69.58)	104 (64.20)	1.51	0.220
	Combined	115 (30.42)	58 (35.80)		
Pulmonary dysfunction	–	247 (65.34)	94 (58.02)	2.61	0.106
	+	131 (34.66)	68 (41.98)		
Vagus nerve injury	–	134 (35.45)	65 (40.12)	0.106	0.302
	+	244 (64.55)	97 (59.88)		
Histology	Benign	29 (7.67)	16 (9.88)	0.72	0.396
	Malignant	349 (92.33)	146 (90.12)		
Cough	–	259 (68.52)	113 (69.75)	0.08	0.776
	+	119 (31.48)	49 (30.25)		
Sputum	–	249 (92.33)	152 (93.83)	0.38	0.537
	+	29 (7.67)	10 (6.17)		

(UL, Upper lobe; NUL, non upper lobe; + = yes; - = no).

**Table 2:** Baseline characteristics of all patients in the training cohort and external validation cohort.

Variables		Training cohort (n=378) M (P25, P75)/N (%)	External validation cohort (n=160) M (P25, P75)/N (%)	t/χ^2^	P
Age		55.36 (49, 63)	56.05 (51, 62)	-0.64	0.524
BMI		23.90 (22.03, 25.81)	23.99 (21.92, 25.81)	-0.32	0.748
Drainage duration		3.63 (2, 5)	3.95 (3, 5)	4.21	0.002
Sex	Male	137 (36.24)	58 (36.25)	0.01	0.999
	Female	241 (63.76)	102 (63.75)		
Underlying disease	–	253 (66.93)	114 (71.25)	0.97	0.325
	+	125 (33.07)	46 (28.75)		
Tobacco exposure	Never	297 (78.57)	122 (76.25)	0.35	0.794
	Former	9 (2.38)	5 (3.13)		
	Current	72 (19.05)	33 (20.63)		
Tumor location	UL	100 (24.46)	38 (23.75)	0.43	0.511
	NUL	278 (73.54)	122 (76.25)		
Type of surgery	Single	263 (69.58)	109 (68.12)	0.11	0.739
	Combined	115 (30.42)	51 (31.88)		
Pulmonary dysfunction	–	247 (65.34)	101 (63.12)	0.24	0.263
	+	131 (34.66)	59 (36.88)		
Vagus nerve injury	–	134 (35.45)	57 (35.62)	0.01	0.969
	+	244 (64.55)	103 (64.38)		
Histology	Benign	29 (7.67)	17 (10.62)	1.25	0.263
	Malignant	349 (92.33)	143 (89.38)		
Cough	–	259 (68.52)	111 (69.38)	0.04	0.845
	+	119 (31.48)	49 (30.62)		
Sputum	–	249 (92.33)	148 (92.50)	0.01	0.945
	+	29 (7.67)	12 (7.50)		

(UL, Upper lobe; NUL, non upper lobe; + = yes; - = no).

### 3.2 Screening for predictive factors

Univariate logistic regression analysis was performed in the training cohort, identifying sex, tobacco exposure, tumor location, type of surgery, vagus nerve injury, and postoperative drainage duration as potential risk factors for persistent postoperative cough. These variables were subsequently included in a multivariate logistic regression analysis.

The results demonstrated that five independent factors were significantly associated with the development of persistent postoperative cough following pulmonary segmentectomy (**Table 3**): tobacco exposure (P = 0.019, OR 0.26, 95% CI 0.08–0.80), tumor location (P = 0.04, OR 2.99, 95% CI 1.41–6.35),type of surgery (P = 0.001, OR 3.49, 95% CI 1.84–6.63), vagus nerve injury (P =0.009, OR 2.49, 95% CI 1.25–4.94), drainage duration (P = 0.001, OR 1.46, 95% CI 1.24–1.71).

**Table 3:** Univariate and multivariate logistic regression analyses of the training cohort.

Variables		P	OR	OR95%CI		P	OR	OR95%CI	
Age		0.527	1.01	0.99	1.02				
BMI		0.356	0.97	0.90	1.04				
Drainage duration		0.001	1.45	1.28	1.64	0.001	1.48	1.29	1.70
Sex	Female	0.02	1.75	1.09	2.80	0.686	0.87	0.43	1.73
	Male								
Underlying disease	+	0.879	1.04	0.65	1.64				
	–								
Tobacco exposure	+	0.001	0.31	0.16	060	0.001	0.19	0.07	0.49
	–								
Tumor location	UL	0.009	2.05	1.19	3.52	0.006	2.36	1.27	4.36
	NUL								
Type of surgery	Combined	0.001	3.56	2.24	5.68	0.001	3.70	2.18	6.29
	Single								
Pulmonary dysfunction	+	0.382	1.22	0.78	1.92				
	–								
Vagus nerve injury	+	0.001	2.56	1.55	4.22	0.008	2.15	1.23	3.76
	–								
Histology	Malignant	0.639	1.22	0.53	2.85				
	Benign								

(UL, Upper lobe; NUL, non upper lobe; + = yes; - = no; OR, odds ratio; CI, confidence interval).

### 3.3 Risk prediction nomogram development

Collinearity diagnostics revealed the following variance inflation factors (VIF): tobacco exposure (VIF = 1.044), tumor location (VIF = 1.052), type of surgery (VIF = 1.011), vagus nerve injury (VIF = 1.025), and drainage duration (VIF = 1.017). These results suggest a low likelihood of multicollinearity among the selected variables.

LASSO was utilized for further variable screening. Initially, continuous variables were standardized using Z-scores, and categorical variables were encoded as dummy variables. Subsequently, 5-fold cross-validation was employed to select the optimal penalty coefficient λ=0.0168. The final model retained five key predictive factors, which is consistent with the variables identified through Logistic regression analysis (see supplementary analysis).

**Figure 1:**
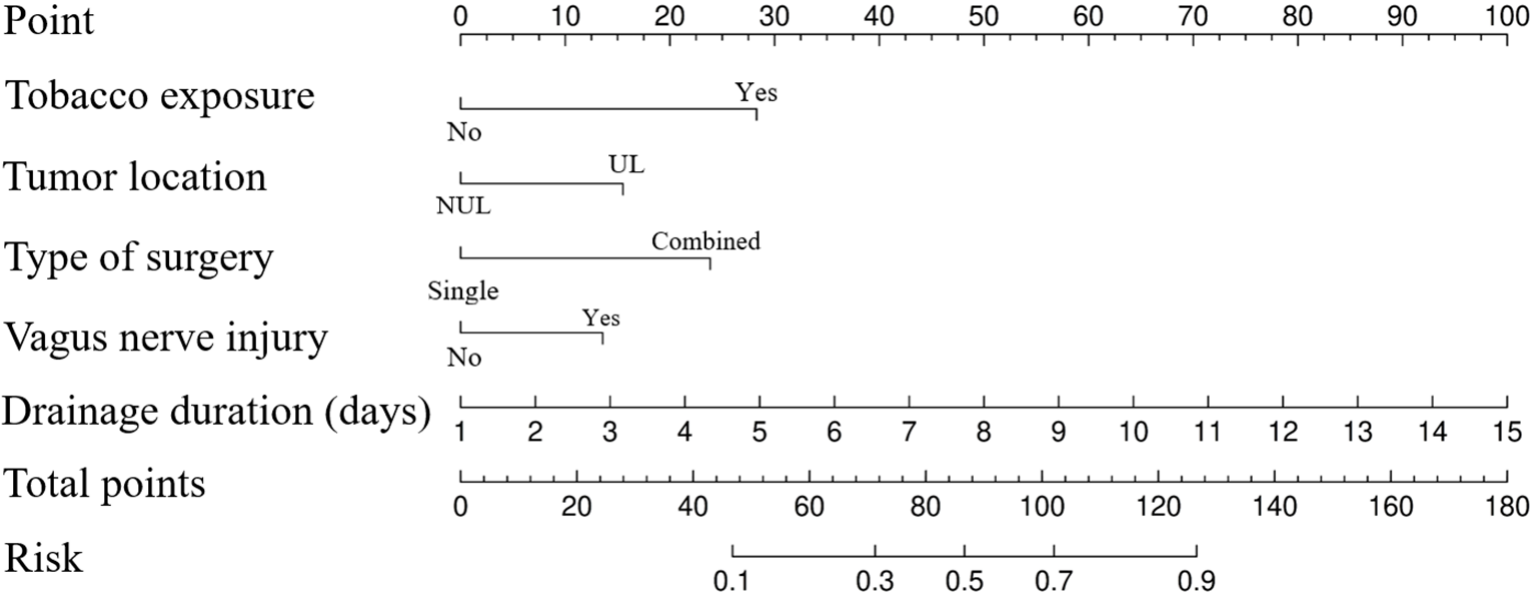
Nomogram for prediction of persistent cough in patients after segmental resection.

Based on these five independent risk factors, a logistic regression model was developed (**Figure 1**). Highertotal scores on the nomogram indicated an increased risk of persistent postoperative cough. Furthermore, the Hosmer-Lemeshow test confirmed a good model fit (P = 0.818).

### 3.4 Predictive accuracy and net benefit of the nomogram

The area under the receiver operating characteristic curve (AUC) for each predictor was as follows: tobacco exposure: 0.58 (95% CI 0.54–0.62), tumor location: 0.56 (95% CI 0.52–0.61), type of surgery: 0.64 (95% CI 0.59–0.69), vagus nerve injury: 0.60 (95% CI 0.55–0.65), drainage duration: 0.68(95% CI 0.62–0.74).

**Figure 2:**
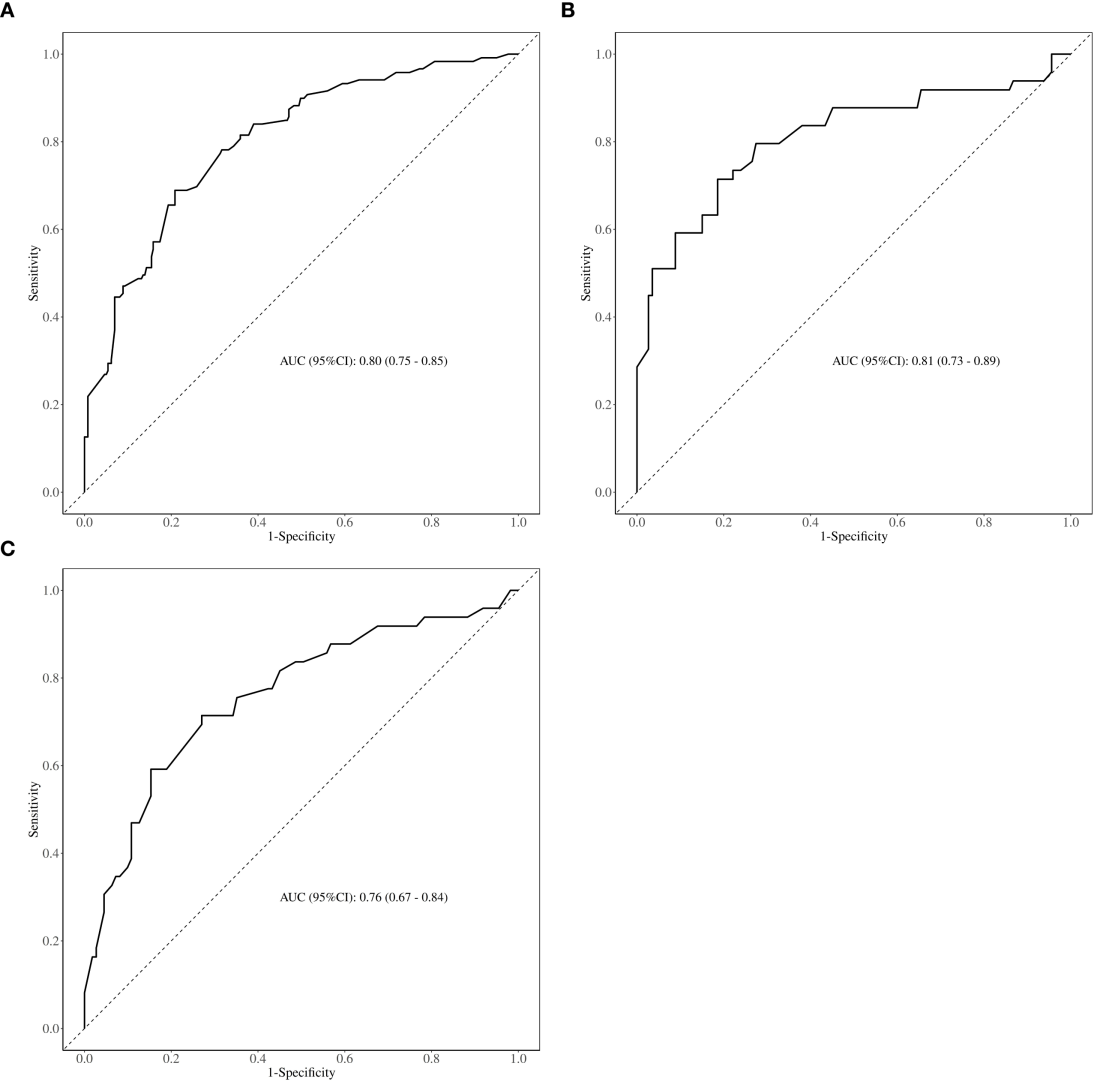
ROC curve. **(A)** Training cohort. **(B)** Internal validation cohort. **(C)** External validation cohort. ROC= Receiver operating characteristics; AUC= Area under ROC curve.

**Figure 3:**
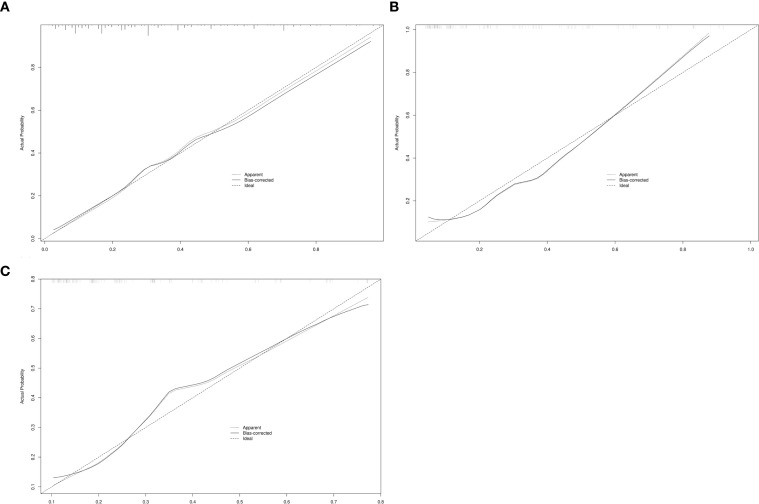
Calibration curve for predicting probability of persistent cough in patients after segmental resection. **(A)**Training cohort. **(B)** Internal validation cohort. **(C)** External validation cohort. (The horizontal axis represents the predicted probability and the vertical axis represents the actual probability).

**Figure 4:**
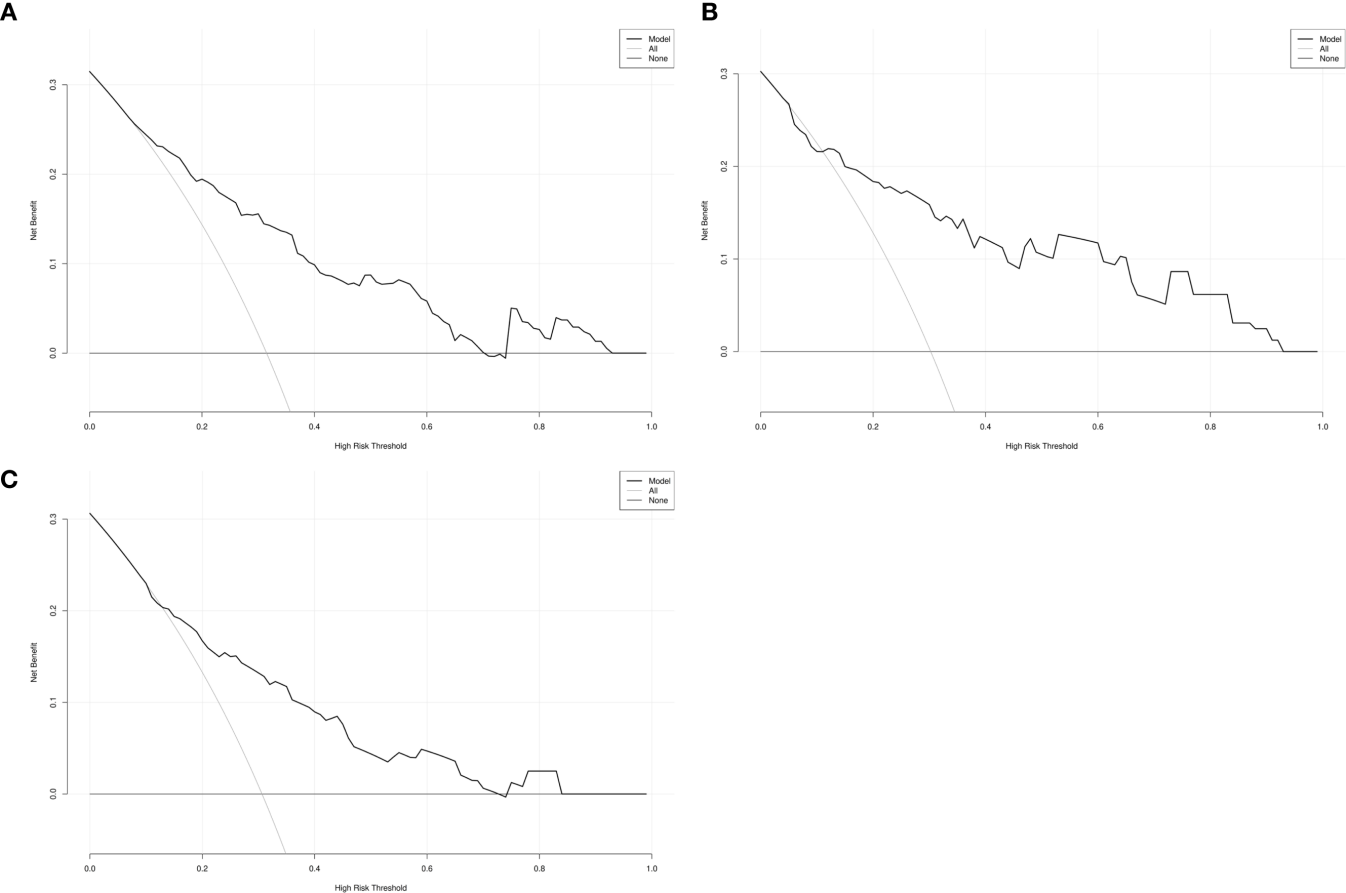
Decision curve analysis in prediction of persistent cough in patients after segmental resection. **(A)** Training cohort. **(B)** Internal validation cohort. **(C)** External validation cohort.

As shown in **Figure 2A**, the AUC of the predictive model in the training cohort was 0.80, with a calibration curve closely aligned with the ideal diagonal line (**Figure 3A**). Moreover, decision curve analysis (DCA) demonstrated a clear net benefit of the predictive model in clinical application (**Figure 4A**).

For internal and external validation, the model achieved an AUC of 0.81 (95% CI 0.73–0.89) and 0.76 (95% CI 0.67–0.84), respectively, indicating good discriminatory ability (**Figures 2B, C**). The model also exhibited strong calibration, as evidenced by a calibration curve closely following the diagonal line (**Figures 3B, C**), and the Hosmer-Lemeshow test showing no significant deviation (P > 0.05). These results suggest a robust predictive performance across different probability thresholds.

Additionally, DCA curves demonstrated a significant net benefit of the predictive model across different threshold probabilities in both the training and validation cohorts (**Figures 4B, C**). Collectively, these findings suggest that the nomogram model holds great potential for clinical decision-making.

## 4 Discussion

Persistent cough is one of the most common and challenging complications following pulmonary resection, with inadequate management potentially progressing to chronic cough (17). This study describes the clinical features and risk factors for persistent postoperative cough following pulmonary segmentectomy. The results indicate that combined segmentectomy, upper lobe segmentectomy, vagus nerve injury, and prolonged postoperative drainage are independent risk factors for the development of persistent cough after surgery, while a history of smoking before surgery appears to be a protective factor. Additionally, we developed a predictive model for this complication, which could support preoperative prevention and intraoperative decision-making. This model allows for risk stratification and individualized interventions for each pulmonary segmentectomy patient, with dynamic postoperative evaluation. High-risk patients can be prioritized for interventions, and relevant departments can be consulted for further treatment. Respiratory therapists and physical therapy resources can be focused on high-risk groups to enhance treatment efficiency, ultimately improving patient adherence to treatment, reducing postoperative cough risk, and lowering healthcare costs.

We found that combined pulmonary segmentectomy is a significant risk factor for persistent cough after surgery. We hypothesize that this may be due to secondary pathological changes resulting from reduced lung volume postoperatively. Combined segmentectomy leads to more severe anatomical and physiological changes in the lung, which could alter airflow dynamics or airway sensitivity, thereby increasing the likelihood of cough (18, 19). However, limited research has focused on this particular factor. Previous studies have demonstrated that patients who undergo lobectomy are more likely to develop postoperative cough than those who undergo sublobar resections (6, 20). One might reasonably infer that the greater the reduction in lung volume, the more severe the secondary changes, and the higher the incidence of postoperative cough. This inference, however, overlooks the varying degrees of mediastinal lymph node dissection between surgical approaches, which is a significant risk factor for postoperative cough. In our study, after controlling for this factor, we confirmed that reduced lung volume is indeed a risk factor for persistent postoperative cough.

As is well known, smoking is a major cause of chronic cough (21), which is why we excluded patients with a pre-existing cough history. Surprisingly, our analysis revealed that tobacco exposure is associated with a reduced risk of cough after pulmonary segmentectomy. In the further analysis, we found that patients currently exposed to tobacco have a lower risk of experiencing persistent coughing after surgery. However, the association may be influenced by unmeasured residual confounding factors. Additionally, other reasons may be explained by the squamous metaplasia of tracheal mucosa caused by long-term smoking, which decreases the sensitivity of cough receptors and thereby reduces the incidence of postoperative cough. This finding is consistent with previous research (22). However, a study by Mariko Fukui and colleagues suggests that smoking cessation can reduce the risk of postoperative complications following lung surgery (23). In our study, none of the patients with a tobacco exposure developed obstructive pneumonia, atelectasis, or other complications, possibly due to the diligent care provided by our nursing team and the use of mechanical sputum clearance.

The vagus nerve plays a crucial regulatory role in the respiratory system, and previous studies have reported that vagus nerve branches are involved in postoperative cough reflex, sputum clearance, and pulmonary immune defense mechanisms (24). Preserving the vagus nerve allows for better adaptation and response to anatomical and physiological changes in the thoracic cavity, reducing cough caused by harmful stimuli. Intraoperative vagus nerve injury significantly increased the incidence of persistent postoperative cough (OR = 2.49). Additionally, the vagus nerve’s esophageal plexus may be connected to the pulmonary branches, and the protective effect of the pulmonary branches could reduce damage to the esophageal plexus, thereby minimizing cough induced by gastroesophageal reflux.

We also assessed the impact of different pulmonary lobe surgeries on postoperative cough incidence, finding that patients undergoing upper lobe surgery were at a higher risk of developing cough than those undergoing resection of other lung lobes. Furthermore, we discovered that longer durations of postoperative closed thoracic drainage were associated with a higher incidence of persistent cough. These risk factors appear to increase the likelihood of persistent cough due to stimulation of lung stretch receptors. In upper lobe segmentectomy, the upper mediastinal lymph nodes are often resected, and the importance of this lymph node dissection in the development of chronic postoperative cough has been previously established (5, 16). Rapidly adapting lung stretch receptors are located around these lymph nodes, and when stimulated, they may induce mechanical irritation (e.g. from cough reflex) or chemical irritation (e.g. from pleural effusion), both of which could trigger rapid adaptation reflexes in the thoracic cavity. In contrast, during non-upper lobe segmentectomy, the upper mediastinal lymph nodes are less frequently addressed, and postoperative cough may occur due to the activation of lower lung stretch receptors as patients begin mobilizing. This hypothesis is supported by a clinical trial that demonstrated that autologous fat grafting to fill postoperative lung cavities reduced the incidence of postoperative cough (11). Additionally, the irritation of the pleura by closed thoracic drainage tubes can reduce respiratory muscle strength, exacerbating postoperative pain, leading to shallow breathing, and thereby increasing the risk of persistent cough (25).

Endotracheal intubation is widely regarded as the most reliable and stable airway management tool. However, existing clinical evidence and related studies indicate that, compared to traditional general anesthesia with endotracheal intubation, anesthesia with preserved spontaneous respiration significantly reduces the incidence of postoperative respiratory complications (26, 27). Moreover, a series of studies and clinical practices conducted by Jianxing He and colleagues have gradually expanded the indications for anesthesia with preserved spontaneous respiration, and their research shows that these patients exhibit better long-term outcomes (28). In the management of pulmonary segmentectomy patients under spontaneous breathing anesthesia at our medical center, we found that the incidence of pulmonary complications was significantly lower in this group compared to the intubation group. However, because the sample size for non-invasive anesthesia was small, we were unable to make a reasonable comparison with the intubation group in this study, and therefore, anesthesia type was not included as a variable in our analysis. This represents a direction for future research. Additionally, comparing preoperative and postoperative bronchoalveolar lavage fluid results in these patients could potentially provide new molecular-level evidence. Despite the clinically significant findings of our study, some limitations remain. First, this is a retrospective study, and therefore, subject to certain limitations. We are working towards conducting a randomized controlled trial to confirm the validity of our conclusions. Second, some meaningful potential influencing factors, such as the patient’s work environment and airway sensitivity, were not assessed due to limitations in the available data. Third, there are some limitations in controlling for confounding factors in this study: (1) the use of bronchodilators may have a bidirectional association with postoperative cough (e.g. patients may use bronchodilators postoperatively due to airway hyperresponsiveness), but the usage data were only based on postoperative records and were not included in baseline assessments; (2) a history of gastroesophageal reflux disease (GERD) was not systematically collected, and GERD may indirectly influence pulmonary outcomes through aspiration. Future studies should adopt a prospective design with improved data collection methods to verify these conclusions. Fourth, persistent postoperative cough is largely attributed to the stimulation of C-fiber nerve fibers (29), and the widespread use of energy devices may increase the excitability of cough receptors, thus raising the incidence of persistent cough postoperatively, which may influence the final results. Fifth, the sample size of the external validation cohort is relatively small, limiting the further external generalizability of the study results. In the future, we will continue to conduct in-depth follow-ups and analyses.

## 5 Conclusion

In conclusion, cough is a common complication in patients following pulmonary segmentectomy and deserves more attention from clinicians. Our study identified combined pulmonary segmentectomy, upper lobe segmentectomy, vagus nerve injury, and prolonged postoperative drainage as independent risk factors for persistent cough, while a history of smoking before surgery served as a protective factor. By using these variables, we constructed a risk prediction model for postoperative cough in pulmonary segmentectomy patients, which was internally validated and confirmed the accuracy and reliability of our findings. The visualized model for risk factors provides clinicians with a simple and intuitive tool for early prediction and intervention of persistent postoperative cough. This is of significant importance for reducing the incidence of cough and improving postoperative quality of life, providing a more reliable basis for the diagnosis and treatment of this patient population.

## Data Availability

The raw data supporting the conclusions of this article will be made available by the authors, without undue reservation.
