# Supplementary material for: Persistent cough after segmental resection, an issue that clinicians need to pay more attention to
Source: Front Oncol. 2025 Aug 8;15:1621841. doi: 10.3389/fonc.2025.1621841 (PMC12370635; doi:10.3389/fonc.2025.1621841)

Table S1: LASSO regression coefficient path map of influence factors.

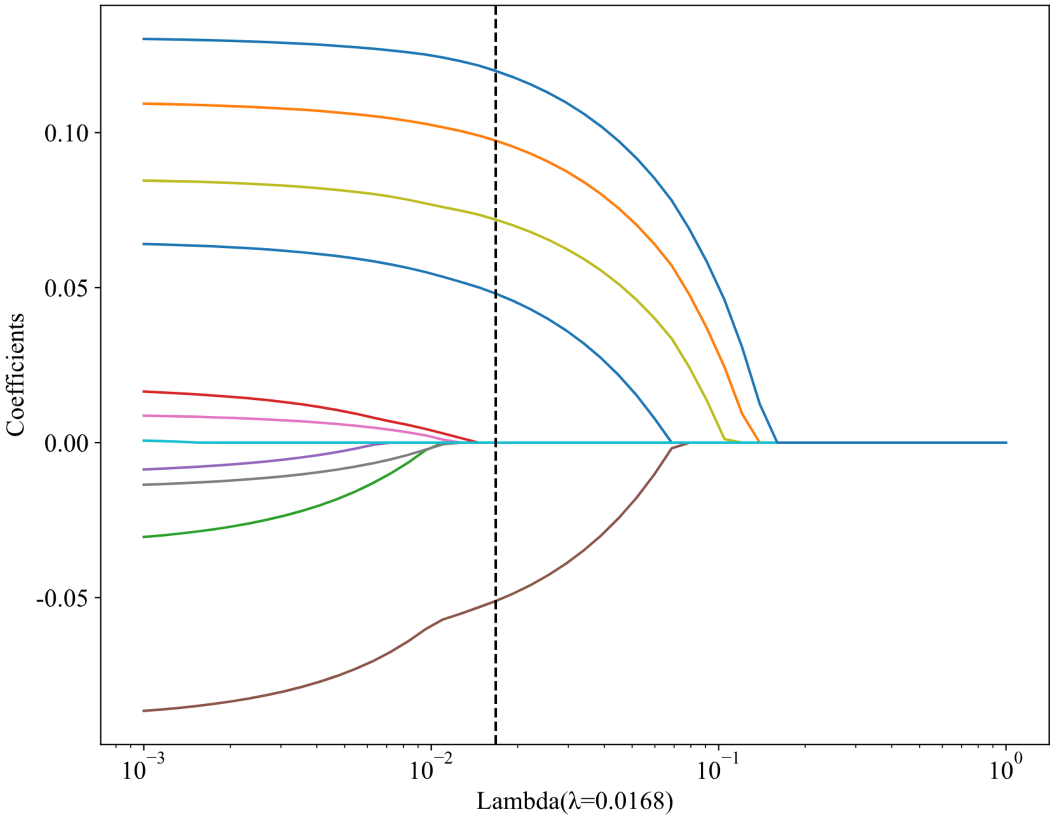

Table S2: Cross validation curve.

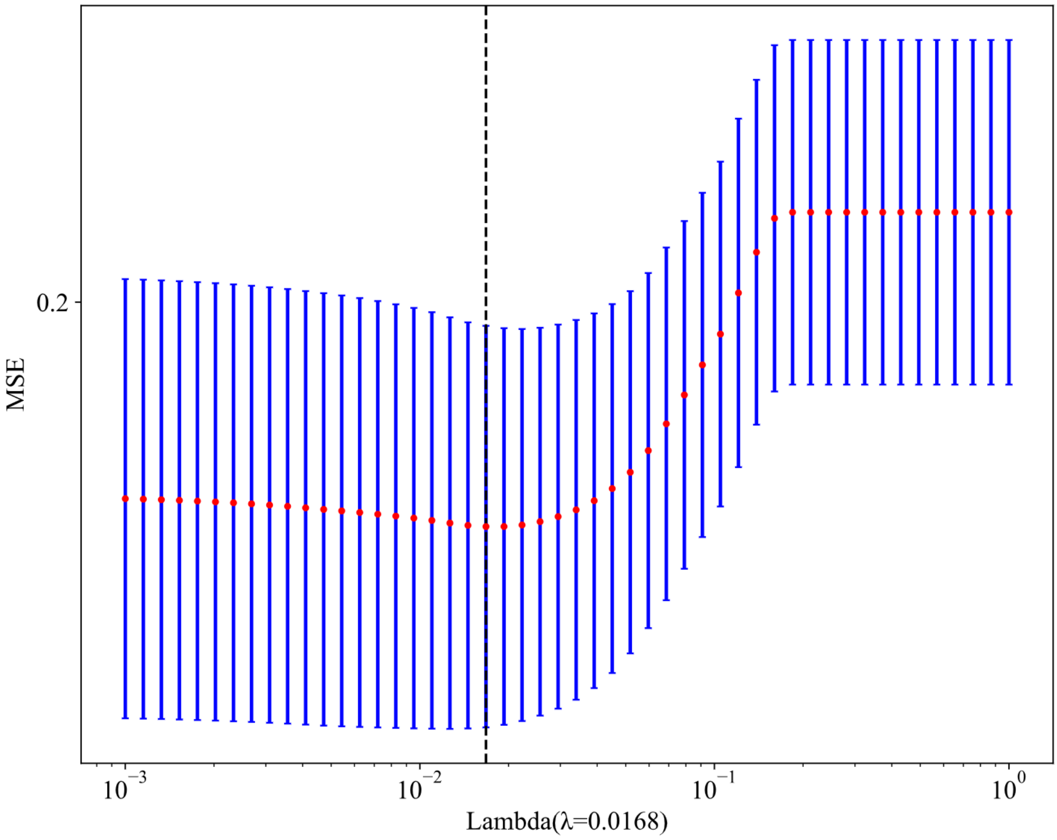

Table S3: Feature selection weight graph.

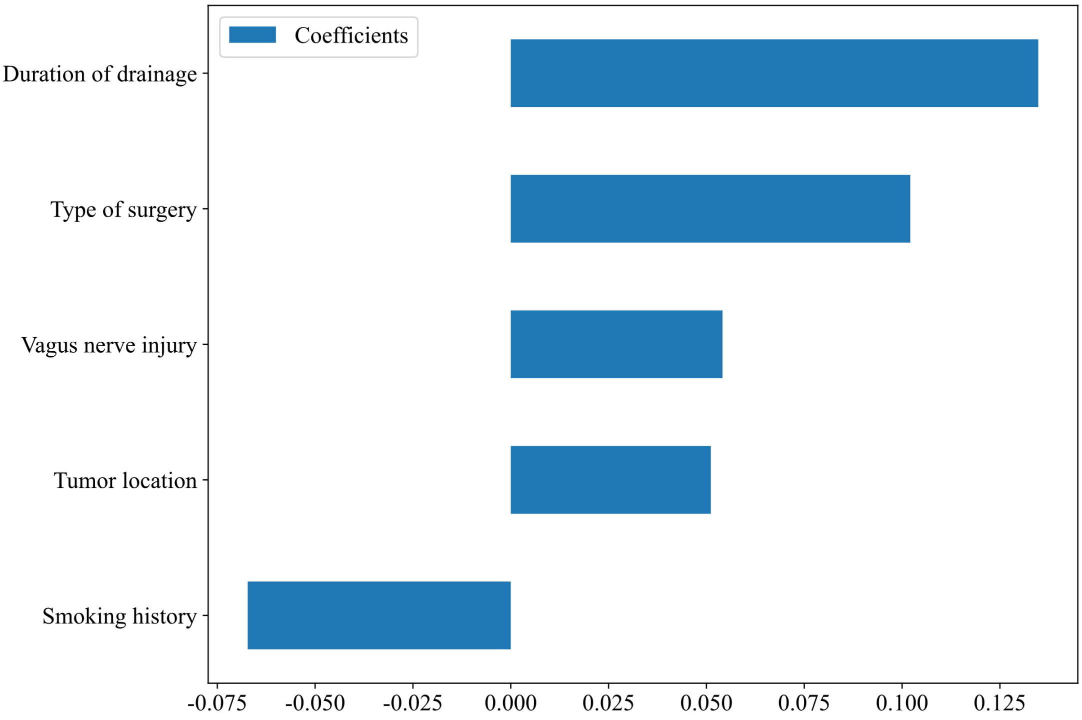

Figure S1: Patients selection.

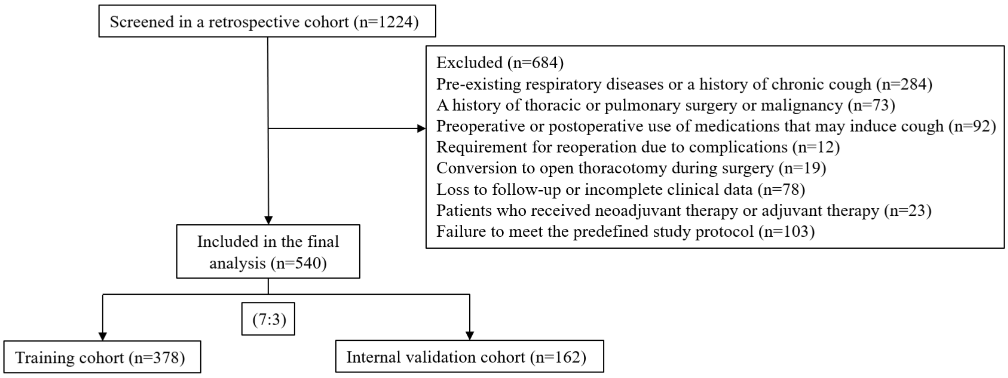

Supplement: Supplementary file 1 [file DataSheet1.pdf]
